# Changes in the microbiota in different intestinal segments of mice with sepsis

**DOI:** 10.3389/fcimb.2022.954347

**Published:** 2023-01-10

**Authors:** Yahui Peng, Jieling Wei, Xiaonan Jia, Feiyu Luan, Mingyin Man, Xiaohui Ma, Yinghao Luo, Yue Li, Nana Li, Qian Wang, Xibo Wang, Yang Zhou, Yuanyuan Ji, Wenjing Mu, Jun Wang, Chunying Wang, Qianqian Zhang, Kaijiang Yu, Mingyan Zhao, Changsong Wang

**Affiliations:** ^1^ Department of Critical Care Medicine, The First Affiliated Hospital of Harbin Medical University, Harbin Medical University, Harbin, China; ^2^ Department of Critical Care Medicine, The Second Affiliated Hospital of Harbin Medical University, Harbin Medical University, Harbin, China; ^3^ Department of Critical Care Medicine, Harbin Medical University Cancer Hospital, Harbin Medical University, Harbin, China

**Keywords:** sepsis, *Muribaculaceae*, *Lachnospiraceae_NK4A136_group*, *Akkermansia*, gut microbiota, mucin-degrading microbes

## Abstract

**Introduction:**

The small intestine, as the main digestion and absorption site of the gastrointestinal tract, is often overlooked in studies, and the overall microbiota does not reflect the makeup of the microbiota in different segments of the intestine. Therefore, we aimed to exclude the influence of routine ICU treatment measures on sepsis patients and observed changes in the diversity and abundance of gut microbiota in different intestinal segments of septic mice.

**Methods:**

The mice were randomly divided into the CLP6h group and the sham group. The contents of the colon and small intestine of the experimental group and the control group were collected after 6 h.

**Results:**

After CLP, the number and structure of the gut microbiota in the colon changed most obviously, among which Bacteroidetes had the most significant changes. *Akkermansia*, *D.Firmicutes_bacterium_M10_2*, *Blautia*, *Bifidobacterium*, *Lactobacillus*, *Candidatus_Arthromitus*, and *Muribaculaceae* were changed in the colon. *Lactobacillus*, *Bifidobacterium*, *Akkermansia*, *Blautia*, *Candidatus_Arthromitus*, and *Lachnospiraceae_NK4A136_group* were changed in the small intestine.

**Discussion:**

Our experiment found that there were different numbers of unique and common gut microbiota in the small intestine and colon after sepsis, and the gut microbiota of the colon changed more drastically after sepsis than the small intestine. Thus, we should focus on protective gut microbiota and mucin-degrading microbes. We hope that these results will provide help for sepsis treatment in the future.

## 1 Introduction

There are billions of bacteria in the intestinal tract, each with the ability to communicate with the host, to digest, store, and redistribute energy and to complete their division within the host, thereby creating a mutually beneficial symbiotic relationship with the host. Dysbiosis of the gut microbiota disrupts this symbiotic balance, leading to the development of host disease (Fay et al., 2017). Existing studies have found that the gut microbiota has a wide range of effects on host disease, ranging from local diseases of the gastrointestinal tract to systemic diseases such as neurological, respiratory, metabolic, liver, and cardiovascular diseases (Lynch and Pedersen, 2016). With an increase in research on the influence of the gut microbiota on the occurrence and development of diseases in the body, many scholars are paying increasing attention to the gut microbiota.

Sepsis is currently one of the major factors affecting human survival and the economy worldwide, and it is the main cause of death in hospitalized patients in the intensive care unit (ICU) (Vincent et al., 2014). Reducing mortality and improving the prognosis of patients with sepsis have long been the major focus of research in critical care medicine. A growing number of researchers are focusing on the gut microbiota of sepsis patients. It has been shown that patients with sepsis have a profoundly distorted gut microbiota composition (Zaborin et al., 2014; Mcdonald et al., 2016; Ojima et al., 2016; Lankelma et al., 2017b). Gut microbiota imbalance plays an important role in the occurrence and development of sepsis and may be an active participant in the development of sepsis (Dickson, 2016; Klingensmith and Coopersmith, 2016). Microbiota dysbiosis has been considered an important factor for increased susceptibility to sepsis (Liu et al., 2020).

Adverse consequences, such as intestinal barrier dysfunction, flora displacement, and immune mechanism disturbances, can be induced by disruptions in the intrinsic balance of the microbial flora caused by routine ICU treatments, such as the administration of antibiotics and proton pump inhibitors. In general, the diversity of gut microbiota in patients with sepsis decreases rapidly upon hospital admission (Mcdonald et al., 2016). In short, these changes in gut microbiota composition can be partially explained by clinical interventions, such as enteral feeding, mechanical ventilation, proton pump inhibitors, opioids, vasopressors, and antibiotics (Vincent et al., 2009; Benus et al., 2010; Dickson, 2016). Moreover, patients with sepsis have impaired gastrointestinal motility and decreased intestinal epithelial integrity, which further damages intestinal epithelium function and allows the expansion and potential translocation of opportunistic pathogens (Donskey, 2004; Haak et al., 2017; Huber-Lang et al., 2018). For example, in an experiment using vancomycin in the treatment of sepsis, it was found that Bifidobacteria were significantly reduced, while several streptococci and lactobacilli were endogenously resistant to vancomycin (Lankelma et al., 2017a). Since interference of the clinical interventions cannot be excluded in the studies of sepsis patients, and the overall microbiota does not reflect the makeup of the microbiota in different segments of the intestine, we chose to design animal experiments to explore the issue of the gut microbiota of sepsis.

In metabolic diseases or gastrointestinal disorders, the small intestine may be the primary site of microbiota related to disease. Compared with the colon, the surface area of the small intestine is greater than 100 times that of the colon, and the mucus layer of the small intestine is much thinner (Johansson et al., 2013). Additionally, the small intestine is the main site for intestinal immune surveillance by lamina propria dendritic cells (Ko and Chang, 2015) and Peyer’s patches (Rios et al., 2016). However, few studies have attempted to characterize the small intestinal microbiota.

Accordingly, we aimed to exclude the influence of routine ICU treatment measures on sepsis patients and observed the changes in the diversity and abundance of gut microbiota in septic mice. Many previous studies have focused on the colon, while the small intestine, as the longest portion of the digestive system with important functions in digestion and absorption, has been overlooked. Therefore, we designed animal experiments to assess the compositions and changes in the gut microbiota in the different intestinal segments.

## 2 Materials and methods

### 2.1 Animals and experimental materials

Specific pathogen-free (SPF)-grade 4-week-old C57BL/6J male mice were provided by Beijing Weitong Lihua Experimental Animal Co., Ltd. (MM-45118M1-96T). All mice were housed in the same cage in a controlled environment (22°C, 12 h light/12 h dark cycle) with free access to food (normal sterile feed) and water. The reason for the use of male mice in experiments is to avoid interference with female estrous cycles, and male mice have better physical health indicators that can guarantee experimental results.

### 2.2 Experimental design

Cecum ligation and puncture (CLP) is a topical method that causes severe abdominal cavity infection of the animal and bacterial and endotoxin release into the bloodstream, causing an inflammatory response and pathological processes that are very similar to clinical sepsis (Rittirsch et al., 2009).

After 3 weeks in the facility (stabilization of gut microbiota), twenty 7-week-old SPF-grade C57BL/6J male mice were selected. The mice were randomly divided into two groups: the CLP6h group (*n* = 10) and the sham group (*n* = 10). We defined “S” as the small intestine group and “C” as the colon group.

Mice in the CLP6h group were opened through the midline of the abdomen under general anesthesia, the cecum was located, and the end one-third of the cecum was ligated with a 4-0 silk thread. The cecum was then punctured with a 5-gauge needle, and a small amount of feces was extruded. Finally, the cecum was returned, and the abdominal cavity was closed. In the sham group, the abdominal cavity was opened for the same amount of time as in the CLP6h group, and then the abdominal cavity was closed. Finally, the mice received an equal amount of fluid resuscitation after surgery.

After CLP, the tight junctions of intestinal epithelial cells and the intercellular distance between adhesion junctions increased significantly and the permeability of the intestinal tract began to peak at 6 h after the induction of sepsis (Yoseph et al., 2016; Obermüller et al., 2020). Therefore, we chose a 6-h time point for sample collection. After 6 h, the contents of the colon and small intestine were collected, while the mice were under general anesthesia, and placed in a −80°C freezer. The fecal samples were extracted from individual mice and stored separately.

### 2.3 HE staining of the small intestine and colon

The intestines were fixed in 4% paraformaldehyde, embedded in paraffin, and processed into 5-µm-thick sections. Routine hematoxylin and eosin staining was performed, and the sections were examined under a confocal microscope (Olympus, Tokyo, Japan) at ×20 magnification.

### 2.4 Microbial community analysis

The corresponding DNA extraction kit (QIAamp Fast DNA Stool Mini Kit, No. 51604) was used to extract genomic DNA from each sample according to the instructions. Then, 1% agarose gel electrophoresis was used to detect the integrity and purity of DNA. DNA concentration and purity were checked using NanoDrop One (Thermo Fisher Scientific, MA, USA). PCR amplification and product electrophoresis detection used genomic DNA as the template and according to the selection of the sequencing region. PCR amplification was performed using primers (Cord: 341F 5′-CCTACGGGRSGCAGCAG-3′ and 806R 5′-GGACTACVVGGGTATCTAATC-3′, Invitrogen, Carlsbad, CA, USA) with barcodes and PremisTaq (TaKaRa Biotechnology, Dalian Co., Ltd., China). One microliter of each primer (10 μM) and 3 μl of DNA (20 ng/μl) template in a volume of 50 μl were amplified by thermocycling as follows: 5 min at 94°C for initialization; 30 cycles of 30 s denaturation at 94°C, 30 s annealing at 52°C, and 30 s extension at 72°C; followed by a 10-min final elongation at 72°C. The PCR instrument was a Bio-Rad S1000 (Bio-Rad Laboratory, CA, USA). After comparing the concentrations of PCR products using Gene Tool Analysis software (Version 4.03.05.0, SynGene, England), the required volume of each sample was calculated according to the principle of equal mass, and the PCR products were mixed. The PCR mixture was recovered using the E.Z.N.A.^®^ Gel Extraction Kit (Omega, USA), and the target DNA fragments were recovered by elution with TE buffer. Subsequent library construction was performed according to the standard process of NEBNext^®^ Ultra™ DNA Library Prep Kit for Illumina^®^ (New England Biolabs, MA, USA). Upon completion, library quality was assessed on a Qubit2.0fluorometer (Thermo Fisher Scientific, MA, USA). Once complete, in-flight sequencing was performed using the high-throughput sequencing platform HiSeq or MiSeq. Base calling is an algorithm that identifies DNA sequences from row images through computer vision and finally generates sequencing reports.

### 2.5 Statistical analyses

The Kruskal−Wallis rank-sum test was used to compare the significant differences among multiple groups, Dunn’s test was used afterward, and the FDR method was used to correct the *p*-value. One-way ANOVA was used to test whether the means of multiple groups of samples were the same, and all two groups were tested *post hoc* using methods such as Scheffe’s test. When the variance between the two groups was equal, Student’s *t*-test was used, but since the samples in this experiment were independent samples, the independent samples *t*-test or the Wilcoxon rank-sum test was selected, and the *p*-value was corrected by various methods. The differences were considered to be significant if *p <*0.05.

## 3 Results

### 3.1 Distribution of gut microbiota in the two mouse groups

**Figure 1:**
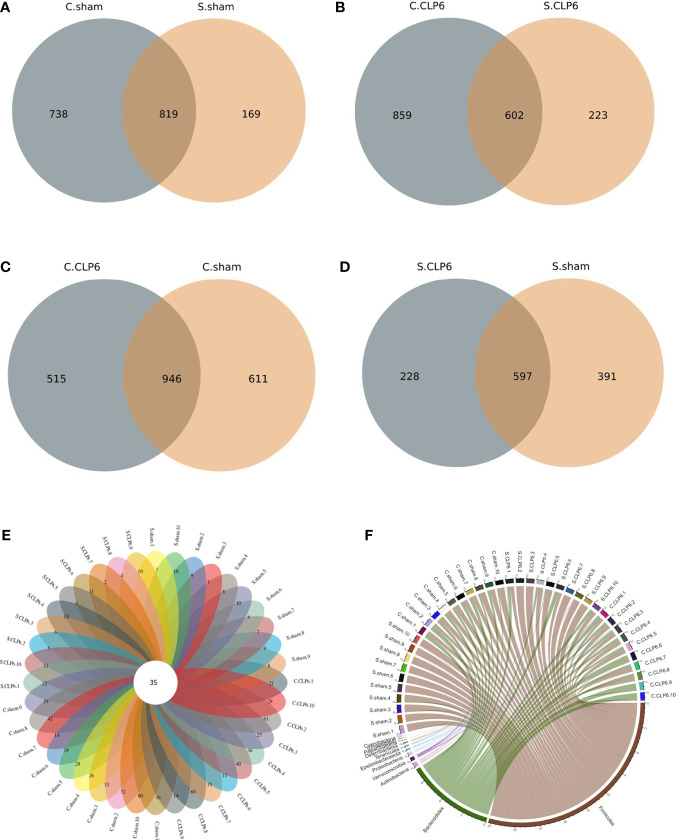
Distribution of gut microbiota in the two mouse groups [CLP6h group (*n* = 10) and the sham group (*n* = 10)]. **(A)** The number of unique gut microbiota species in the colon and small intestine in the sham group. **(B)** The number of unique gut microbiota species in the colon and small intestine in the CLP6h group. **(C)** The number of unique gut microbiota species in the colon in the sham group and CLP6h. **(D)** The number of unique gut microbiota species in the small intestine in the sham group and CLP6h. **(E)** The number of unique gut microbiota for every sample. The cross-section is the number of gut microbiota in common for every sample. **(F)** A chord diagram of the gut microbiota in the intestine of each sample. The circle diameter indicates the number of points; the greater the number is, the larger the diameter of the circle.

The sham group had 169 gut microbiota species unique to the small intestine, 738 gut microbiota species unique to the colon, and 819 gut microbiota species in common (**Figure 1A**). The CLP6h group had 223 gut microbiota species unique to the small intestine, 859 gut microbiota species unique to the colon, and 602 gut microbiota species in common (**Figure 1B**). The colon had 611 gut microbiota species unique to the sham group, 515 gut microbiota species unique to the CLP6h group, and 946 gut microbiota species in common (**Figure 1C**). The small intestine had 391 gut microbiota species unique to the sham group, 228 gut microbiota species unique to the CLP6h group, and 597 gut microbiota species in common (**Figure 1D**).

The gut microbiota was shared to varying levels between each experimental mouse, with 35 gut microbiota species present regardless of the physiological state of the mice (**Figure 1E** **;** **Table 1**). At the phylum level, Firmicutes, Bacteroidetes, and Actinobacteria were the main phyla (**Figure 1F**).

**Table 1:** The 35 common gut microbiota in different segments of the intestine.

OUT ID	Kingdom	Phylum	Class	Order	Family	Genus	Species
**OUT_11**	Bacteria	Bacteroidetes	Bacteroidia	Bacteroidales	Muribaculaceae	–	–
**OTU_10**	Bacteria	Firmicutes	Erysipelotrichia	Erysipelotrichales	Erysipelotrichaceae	*Dubosiella*	*Firmicutes_bacterium_M10_2*
**OTU_114**	Bacteria	Actinobacteria	Coriobacteriia	Coriobacteriales	Eggerthellaceae	*Enterorhabdus*	Uncultured_bacterium
**OTU_12**	Bacteria	Bacteroidetes	Bacteroidia	Bacteroidales	Muribaculaceae	–	–
**OTU_122**	Bacteria	Actinobacteria	Coriobacteriia	Coriobacteriales	Eggerthellaceae	*Enterorhabdus*	Uncultured_bacterium
**OTU_14**	Bacteria	Bacteroidetes	Bacteroidia	Bacteroidales	Muribaculaceae	–	–
**OTU_15**	Bacteria	Bacteroidetes	Bacteroidia	Bacteroidales	Muribaculaceae	–	–
**OTU_1**	Bacteria	Firmicutes	Bacilli	Lactobacillales	Lactobacillaceae	*Lactobacillus*	–
**OTU_186**	Bacteria	Bacteroidetes	Bacteroidia	Bacteroidales	Muribaculaceae	–	–
**OTU_18**	Bacteria	Firmicutes	Clostridia	Clostridiales	Lachnospiraceae	*Blautia*	–
**OTU_19**	Bacteria	Firmicutes	Erysipelotrichia	Erysipelotrichales	Erysipelotrichaceae	*Allobaculum*	Uncultured_bacterium
**OTU_20**	Bacteria	Actinobacteria	Actinobacteria	Bifidobacteriales	Bifidobacteriaceae	*Bifidobacterium*	–
**OTU_21**	Bacteria	Bacteroidetes	Bacteroidia	Bacteroidales	Muribaculaceae	–	–
**OTU_2**	Bacteria	Firmicutes	Bacilli	Lactobacillales	Lactobacillaceae	*Lactobacillus*	–
**OTU_24**	Bacteria	Firmicutes	Clostridia	Clostridiales	Lachnospiraceae	*Anaerostipes*	Uncultured_bacterium
**OTU_27**	Bacteria	Firmicutes	Erysipelotrichia	Erysipelotrichales	Erysipelotrichaceae	*Faecalibaculum*	Uncultured_bacterium
**OTU_28**	Bacteria	Bacteroidetes	Bacteroidia	Bacteroidales	Muribaculaceae	–	–
**OTU_30**	Bacteria	Bacteroidetes	Bacteroidia	Bacteroidales	Muribaculaceae	–	–
**OTU_35**	Bacteria	Firmicutes	Clostridia	Clostridiales	Lachnospiraceae	*Blautia*	Uncultured_bacterium
**OTU_36**	Bacteria	Firmicutes	Clostridia	Clostridiales	Lachnospiraceae	Uncultured	–
**OTU_37**	Bacteria	Firmicutes	Clostridia	Clostridiales	Lachnospiraceae	*Lachnospiraceae_NK4A136_group*	–
**OTU_38**	Bacteria	Firmicutes	Clostridia	Clostridiales	Lachnospiraceae	–	–
**OTU_42**	Bacteria	Firmicutes	Clostridia	Clostridiales	Ruminococcaceae	*Anaerotruncus*	Uncultured_bacterium
**OTU_43**	Bacteria	Bacteroidetes	Bacteroidia	Bacteroidales	Muribaculaceae	–	–
**OTU_44**	Bacteria	Firmicutes	Clostridia	Clostridiales	Lachnospiraceae	*Lachnospiraceae_NK4A136_group*	–
**OTU_45**	Bacteria	Bacteroidetes	Bacteroidia	Bacteroidales	Muribaculaceae	–	–
**OTU_4**	Bacteria	Firmicutes	Bacilli	Lactobacillales	Lachnospiraceae	*Lactobacillus*	–
**OTU_47**	Bacteria	Firmicutes	Clostridia	Clostridiales	Lachnospiraceae	*Lachnospiraceae_NK4A136_group*	*Lachnospiraceae_bacterium_AJ110941*
**OTU_48**	Bacteria	Firmicutes	Clostridia	Clostridiales	Lactobacillaceae	A2	*Lachnospiraceae_bacterium_538*
**OTU_5**	Bacteria	Bacteroidetes	Bacteroidia	Bacteroidales	Muribaculaceae	–	–
**OTU_52**	Bacteria	Bacteroidetes	Bacteroidia	Bacteroidales	Muribaculaceae	–	–
**OTU_58**	Bacteria	Firmicutes	Clostridia	Clostridiales	Lachnospiraceae	Uncultured	–
**OTU_6**	Bacteria	Firmicutes	Clostridia	Clostridiales	Clostridiaceae_1	*Candidatus_Arthromitus*	–
**OTU_9**	Bacteria	Firmicutes	Bacilli	Lactobacillales	Lactobacillaceae	*Lactobacillus*	–
**OTU_66**	Bacteria	Firmicutes	Clostridia	Clostridiales	Ruminococcaceae	*Anaerotruncus*	–

### 3.2 Changes in specific bacterial species in different intestinal segments

**Figure 2:**
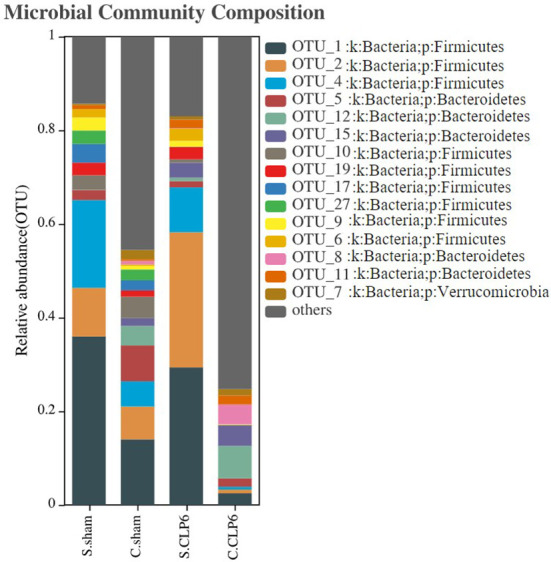
Histogram of gut microbiota distribution in four groups. The abscissa represents the groupings, and the ordinate represents the gut microbiota abundance values. The taxa with an abundance above 1% were selected, and all abundances were in the top 15 for classification.

At the phylum level, Firmicutes, Bacteroidetes, and Verrucomicrobia were the main phyla in the small intestine of the sham group, and Firmicutes, Bacteroidetes, Actinobacteria, and Verrucomicrobia were the main phyla in the colon of the sham group. However, after CLP, the abundance of Firmicutes and Actinobacteria decreased, while the abundance of Bacteroidetes, Proteobacteria, and Epsilonbacteraeota increased. The abundance of Verrucomicrobia was increased in the small intestine and was decreased in the colon after CLP (**Figure 2**). The Firmicutes/Bacteroidetes ratio (F/B ratio) was determined. In the sham group, the F/B ratio was 16.75 in the small intestine and 1.69 in the colon. In the CLP6h group, the F/B ratio was 4.69 in the small intestine and 0.89 in the colon. The F/B ratio in the small intestine was bigger than that in the colon. After CLP, the F/B ratio was decreased (**Supplementary Table S1**).

**Figure 3:**
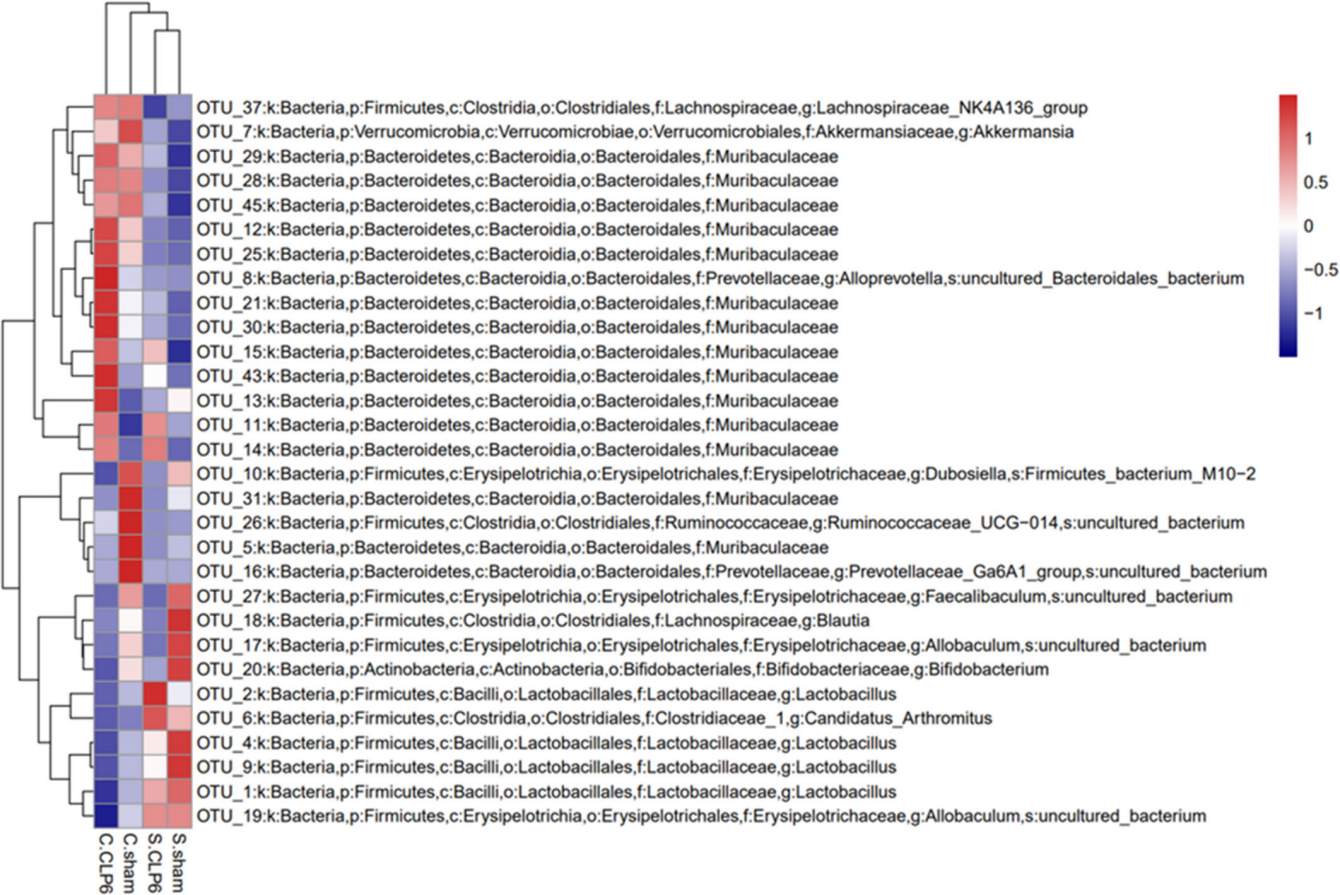
The dominant gut microbiotas in the different intestinal segments of mice in the sham group and the CLP6h group were characterized by their abundances in a heatmap. Abundance is represented by color depth. The redder the color of the square, the higher the abundance of the strain among the samples.

Changes in the species levels in the small intestine and colon before and after sepsis were as follows: *Lactobacillus*, *Bifidobacterium*, and *Blautia* were the dominant microbiota in the small intestine of the sham group. *Lactobacillus* and *Candidatus_Arthromitus* were the dominant microbiota in the small intestine of the CLP6h group. Looking specifically at the small intestine, we observed the following: *Lactobacillus*, *Bifidobacterium*, *Blautia*, and *Lachnospiraceae_NK4A136_group* were decreased after CLP, while *Candidatus_Arthromitus* and *Akkermansia* were increased after CLP. The colon in the sham group was dominated by *Akkermansia* and *Muribaculaceae*, whereas the colon in the CLP6h group was dominated by *Muribaculaceae*. Looking specifically at the colon, we made the following observations: *Akkermansia*, *D.firmicutes-bacterium-M10-2*, *Blautia*, *Bifidobacterium*, *Lactobacillus*, and *Candidatus_Arthromitus* were reduced after CLP, while *Muribaculaceae* and *Alloprevotella* were increased (**Figure 3**).

### 3.3 Changes in the composition of the microbiota in different intestinal segments

**Figure 4:**
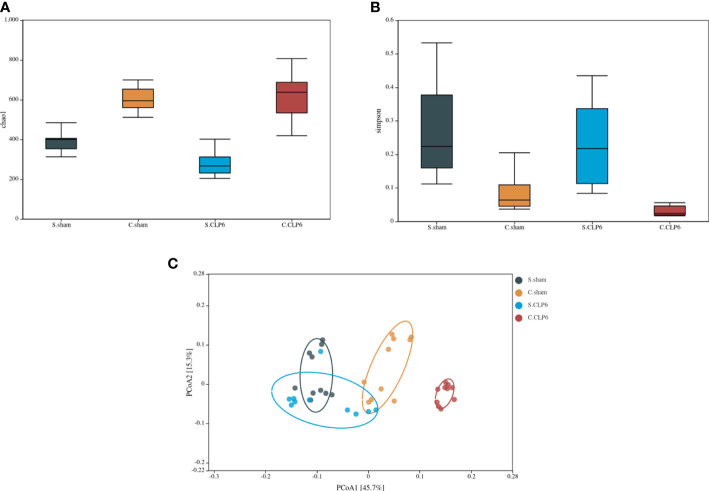
Changes in the composition of the microbiota in different intestinal segments. **(A)** Comparison of gut microbiota richness in the small intestine and colon before and after sepsis. **(B)** Comparison of gut microbiota diversity of the colon and small intestine before and after sepsis. **(C)** PCoA analysis. The dots with different colors represent different sample groups. The closer the spatial distance of the sample is, the more similar the species composition structure of the sample is.

Analysis of the alpha richness revealed the following: The average richness of the small intestine microbiota was lower than that of the colon microbiota. The average richness of the colon microbiota in the CLP6h group was significantly richer than that in the sham group. The average richness of the small intestine microbiota in the CLP6h group was significantly lower than that in the sham group (**Figure 4A**).

Alpha diversity analysis revealed the following: The small intestine microbiota diversity was smaller than that in the colon in the two groups. The diversity of the small intestine microbiota in the CLP6h group was greater than that in the sham group. The diversity of the colon microbiota in the CLP6h group was greater than that in the sham group (**Figure 4B**).

**Figure 5:**
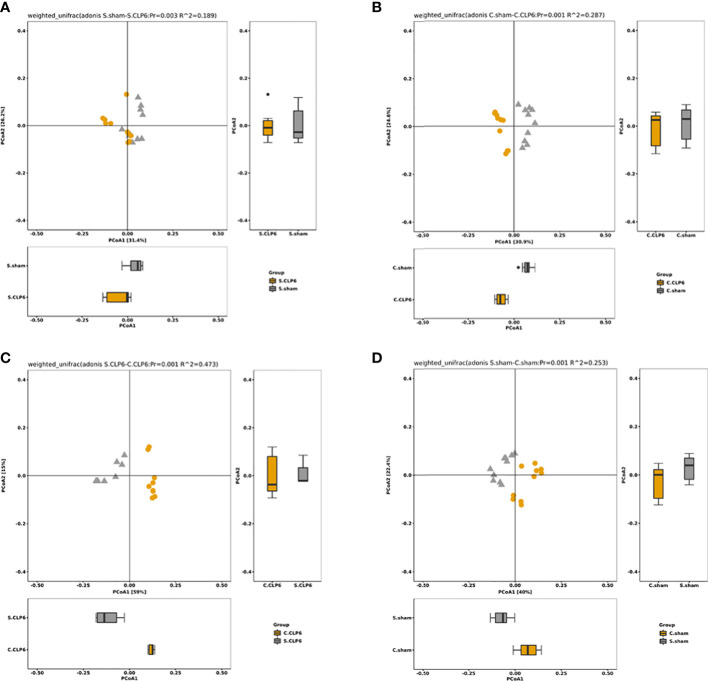
The OTU PCA and PCoA weighted analysis of the difference in gut microbiota. Weighted UniFrac was included in the calculation on the basis of UniFrac to distinguish differences in species abundance. **(A)** Species abundance in the small intestine of the sham group and the CLP6h group was significantly different (*p* = 0.003). **(B)** Species abundance in the colon of the sham group and the CLP6h group was significantly different. (*p* = 0.001). **(C)** In the CLP6h group, the composition of the microbiota in the small intestine and colon was significantly different (*p* = 0.001). **(D)** In the sham group, the composition of the microbiota in the small intestine and colon was significantly different (*p* = 0.001).

Through beta diversity analysis, we found that the composition of the small intestine microbiota differed from that of the colon microbiota in the two groups. After CLP6h, the small intestine microbiota was relatively similar in the two groups, while the composition of the colon microbiota differed from the sham group (**Figure 4C**). A weighted principal coordinate analysis (PCoA2) based on the UniFrac algorithm showed significant differences between pairwise comparisons in the small intestine and the colon after CLP (*p* = 0.001) (**Figure 5**).

### 3.4 Changes in the abundance of the microbiota before and after CLP

**Figure 6:**
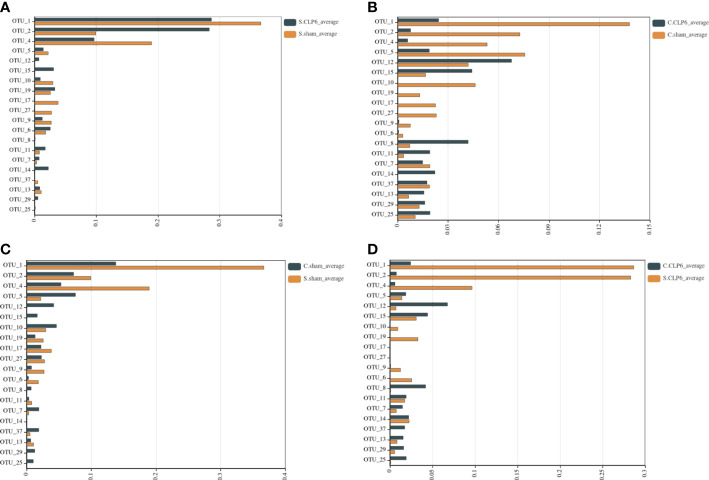
Comparison of gut microbiota in different intestinal segments of the two groups. The abscissa represents the abundance values, and the ordinate represents the bacterial groups. The Wilcoxon signed-rank sum test was used. OTU_1, OTU_2, OTU_4, and OTU_9—k: Bacteria, p: Firmicutes, c: Bacilli, o: Lactobacillales, f: Lactobacillaceae, g: *Lactobacillus*. OTU_5, OTU_12, OTU_15, OTU_11, OTU_14, OTU_13, OTU_29, and OTU_25—k: Bacteria, p: Bacteroidetes, c: Bacteroidia, o: Bacteroidales, f: *Muribaculaceae*. OTU_10—k: Bacteria, p: Firmicutes, c: Erysipelotrichia, o: Erysipelotrichales, f: Erysipelotrichaceae, g: *Dubosiella*, s: *Firmicutes_bacterium_M10_2*. OTU_19, OTU_17, and OTU_27—k: Bacteria, p: Firmicutes, c: Erysipelotrichia, o: Erysipelotrichales, f: Erysipelotrichaceae, g: Allobaculum, s: uncultured_bacterium. OTU_6—k: Bacteria, p: Firmicutes, c: Clostridia, o: Clostridiales, f: Clostridiaceae_1, g: *Candidatus_Arthromitus*. OTU_8—k: Bacteria, p: Bacteroidetes, c: Bacteroidia, o: Bacteroidales, f: Prevotellaceae, g: Alloprevotella, s: uncultured_Bacteroidales_bacterium. OTU_7—k: Bacteria, p: Verrucomicrobia, c: Verrucomicrobiae, o: Verrucomicrobiales, f: Akkermansiaceae, g: *Akkermansia*. OTU_37: k: Bacteria, p: Firmicutes, c: Clostridia, o: Clostridiales, f: Lachnospiraceae, g: *Lachnospiraceae_NK4A136_group*. **(A)** Comparison of gut microbiota in the small intestine of the sham group and the CLP6h group. **(B)** Comparison of gut microbiota in the colon of the sham group and the CLP6h group. **(C)** Comparison of gut microbiota between the small intestine in the sham group and the colon in the sham group. **(D)** Comparison of gut microbiota between the small intestine of the CLP6h group and the colon of the CLP6h group.

In the small intestine, the comparison found that *Muribaculaceae* were not present in the sham group; however, this type of bacteria appeared in the CLP6h group. Compared with the sham group, *Lachnospiraceae_NK4A136_group* and *Lactobacillales* disappeared completely in the CLP6h group (**Figure 6A**; **Supplementary Table S2**).

In the colon, the abundance of *Lactobacillales* in the CLP6h group was lower than that in the sham group. The abundance of *Muribaculaceae* in the CLP6h group was significantly higher than that in the sham group. Compared with the sham group, *Erysipelotrichales* disappeared completely in the CLP6h group (**Figure 6B**; **Supplementary Table S3**).

### 3.5 Changes in the abundance of the microbiota in different intestinal segments

In the sham group, the abundance of *Lactobacillales* in the small intestine was significantly higher than that in the colon. The abundance of *Bacteroidales* in the small intestine was significantly lower than that in the colon. *Erysipelotrichales* varied in abundance in the small intestine and colon (**Figure 6C**; **Supplementary Table S4**).

In the CLP6h group, *Lactobacillales* was higher in the small intestine than in the colon. *Bacteroidales* was lower in the small intestine than in the colon. Compared with the colon, *Erysipelotrichales* was still partially present in the small intestine (**Figure 6D**; **Supplementary Table S5** **)**.

### 3.6 Histological examination of the small intestine and colon

**Figure 7:**
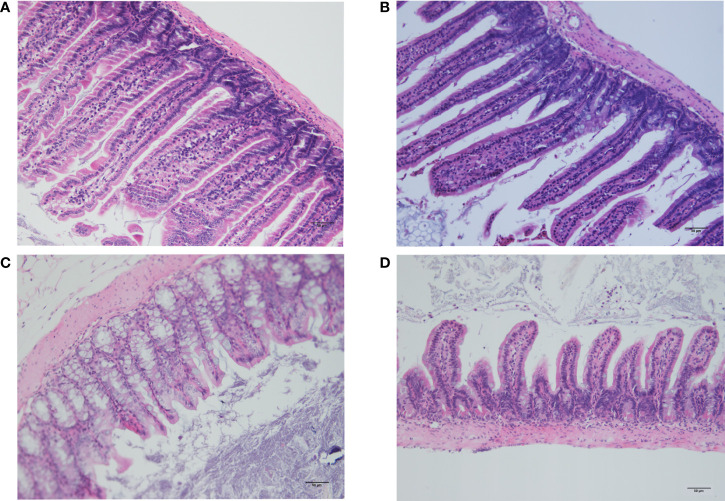
Histological examination of the small intestine and colon. **(A)** Histological analysis of the small intestine in the sham group. The intestinal mucosal villi were unchanged and normal. **(B)** Histological analysis of the small intestine in the CLP6h group. The intestinal mucosal villi were sparse and irregular, and the villi became short and broken, with reduced mucosal layer glands and infiltration of inflammatory cells in the muscularis in the CLP6h group. **(C)** Histological analysis of the colon in the sham group. The intestinal mucosal villi were unchanged and normal. **(D)** Histological analysis of the colon in the CLP6h group. The intestinal mucosal villi were sparse and irregular, and the villi became short and broken, with vacuolization at the top, reduced mucosal layer glands, and infiltration of inflammatory cells in the muscularis in the CLP6h group.

Histological analysis of the small intestine and colon using HE revealed that the intestinal mucosal villi were sparse and irregular and the villi became short and broken, with vacuolization at the top, reduced mucosal layer glands, and infiltration of inflammatory cells in the muscularis in the CLP6h group (**Figure 7**). It was clearly observed that intestinal damage appeared in the CLP6h group.

## 4 Discussion

Our experiment found that the gut microbiota in different intestinal segments of mice with sepsis changes. There were different numbers of unique and common gut microbiota in the small intestine and colon before sepsis. In the sham group, *Lactobacillus*, *Bifidobacterium*, and *Blautia* were the dominant microbiota in the small intestine, and *Akkermansia* and *Muribaculaceae* were the dominant microbiota in the colon. After sepsis, the gut microbiota of both the small intestine and colon changed. In the small intestine, *Lactobacillus*, *Bifidobacterium*, *Blautia*, and *Lachnospiraceae_NK4A136_group* were decreased, while *Candidatus_Arthromitus* and *Akkermansia* were increased. In the colon, *Akkermansia*, *D.firmicutes-bacterium-M10-2*, *Blautia*, *Bifidobacterium*, *Lactobacillus*, and *Candidatus_Arthromitus* were reduced, while *Muribaculaceae* and *Alloprevotella* were increased.

In our results, the different intestinal segments had different gut microbiota in the same group. First, this finding may be related to the different physiological functions of the different intestinal segments. Studies have shown that the composition of gut microbiota differs in a location-specific manner. The abundance of Firmicutes gradually decreases from the stomach to feces, while the abundance of Bacteroidetes gradually increases in healthy mice (Lkhagva et al., 2021). These results agree with our results in the sham group. Under normal physiological conditions, due to the anatomical characteristics of the intestine itself and the differences in ion composition, pH, water content, and physiological functions in each intestinal segment, there are differences in the richness and diversity of the gut microbiota species in different intestinal segments (Moran and Jackson, 1992; El Aidy et al., 2015; Dickson, 2016). Changes in the gut microbiota occur in response to changes in the intestinal environment. Na+ and pH play a key role in shaping the gut microbiota. Meanwhile, NHE3 (Na^+^/H^+^-exchanger isoform 3) ion transport also plays a key role. It not only regulates the intestinal environment but also establishes bacterial niches (Engevik et al., 2013; Larmonier et al., 2013). Second, there are differences in the digestion of nutrients between the colon and small intestine. Some polysaccharides are resistant to digestion in the small intestines and enter the colon where they provide substrates for the complex gut microbiota that resides there (Kiela and Ghishan, 2016; Rastall et al., 2022). Next, there are significant differences in the mucus composition in different intestinal segments. The small intestine has a single unattached mucus layer, and the colon has two layers of mucus. The inner layer is attached and impervious to bacteria. The outer layer is less dense and unattached and is the habitat for commensal bacteria (Johansson et al., 2011; Johansson et al., 2013).

After sepsis, the gut microbiota changes in the different intestinal segments. At the phylum level, the abundance of Firmicutes was decreased, while the abundance of Bacteroidetes was increased. An increase in Bacteroidetes members may be positively correlated with disease onset and can induce colitis in antibiotic-pretreated mice (Schwab et al., 2014).

In a probiotic experiment in mice with sepsis, *Lactobacillus* reduced intestinal apoptosis, promoted epithelial cell proliferation, and reduced the expression of systemic and local inflammatory factors, thereby reducing the mortality rate of sepsis (Khailova et al., 2013). *Bifidobacterium* inhibits harmful bacteria to improve intestinal barrier function; it can regulate intestinal immune homeostasis to protect against harmless antigens and bacteria by changing the function of dendritic cells, or it can take targeted protective measures against pathogens (Azad et al., 2018). *Blautia* inhibits colonization of the intestinal tract by pathogenic bacteria by producing bacteriocins, thereby affecting the composition of the intestinal microbiota and regulating the composition of the microbiota (Yang et al., 2021). The three protective gut microbiota constituents *Lactobacillus*, *Bifidobacterium*, and *Blautia* were decreased in all intestinal segments after sepsis in our experiments.

The most important aspect of intestinal health is the state of balanced richness and variety of gut microbiota. *Akkermansia* is a mucin-degrading microbe in the gut. The intestinal mucus barrier is the body’s first line of defense against bacteria. Mucins are an important component of the mucus layer of the intestinal epithelium. Mucins form a transparent mucus layer on the surface of the intestinal tissue and are the major sites where a large number of intestinal microorganisms inhabit (Paone and Cani, 2020). Low *Akkermansia muciniphila* in the gut may indicate a thinner mucus layer, thus weakening intestinal barrier function, in addition to increased translocation of bacteria which tends to be lower in patients with inflammatory bowel disease, obesity, and type II diabetes. Excessive *Akkermansia* will survive the excessive consumption of mucin, a survival advantage that most other bacteria lack (Geerlings et al., 2018; Zhang et al., 2019). Interesting phenomena were found in our experimental results; *Akkermansia* was decreased in the colon but increased in the small intestine. We suspect that in the intestine of septic mice, *Akkermansia* without balance can damage the intestinal barrier, thus aggravating sepsis.

Meanwhile, there were also two other mucin-degrading microbes in our results. *Muribaculaceae* and *Alloprevotella* were increased in the colon after CLP. Mucin-degrading microbes are known to harbor glycosyl hydrolases (GHs) which cleave specific glycan linkages (Glover et al., 2022). Because the cecum was ligated at the CLP, it was no longer a normal condition for peristalsis. When cecal content no longer feeds into the colon normally, the nutrients available to the microbiota may be reduced. However, this situation does not affect the survival of these three mucus-degrading microbes. In contrast, they will further increase. This conjecture coincides with our results. In this case, the richness of non-mucin-degrading species is significantly reduced, resulting in reduced species diversity, which may lead to intestinal barrier damage. Excessive mucus degradation can thin the mucus layer and induce intestinal inflammation and increased entry of LPS into the bloodstream, making mice susceptible to infectious diseases (Desai et al., 2016; Ormerod et al., 2016; Cannon et al., 2020).

The *Lachnospiraceae_NK4A136_group* bacteria in the gastrointestinal tract produce butyrate and other short-chain fatty acids (SCFAs) by hydrolyzing starch and other sugars, which directly interact with the host’s immune system and regulate the surrounding microbial environment (Vacca et al., 2020). SCFAs are not only the main energy source of the colon but also responsible for intestinal epithelial protection and the regulation of inflammatory intestinal responses, favoring mucus synthesis and upregulating tight junction proteins (Iacob and Iacob, 2019). In our experiments, we found a significant decrease in the *Lachnospiraceae_NK4A136_group* in the small intestine but not in the colon after CLP. Therefore, the *Lachnospiraceae_NK4A136_group* decrease in the small intestine is mainly affected by sepsis. Sepsis affects the richness and diversity of the gut microbiota (Haak and Wiersinga, 2017).

There are some limitations in our experiment. Our experiments yielded only a few phenotypic results, but no mechanistic studies were performed. We have not yet explored the mechanism of the relationship between different gut microbiota constituents in different intestinal segments and sepsis after CLP. This will be our future research direction.

## 5 Conclusion

Overall, this study provides the first insights into comparing the gut microbiota of the different intestinal segments after sepsis in mice. We found that the gut microbiota of the colon changed more drastically after sepsis than the small intestine. Specifically, we should focus on protective gut microbiota (*Lactobacillus*, *Bifidobacterium*, and *Blautia)* and mucin-degrading microbes (*Muribaculaceae* and *Alloprevotella*). We hope that these results will provide help for sepsis treatment in the future.

## Data availability statement

The datasets presented in this study can be found in online repositories. The name of the repository and accession number can be found below: NCBI; PRJNA844197.

## Ethics statement

The animal study was reviewed and approved by the Ethics Committee of the First Affiliated Hospital of Harbin Medical University.

## Author contributions

All authors participated in the design, interpretation of the studies and analysis of the data, and review of the manuscript. KY, ChaW, MZ, and YP designed the research and wrote the manuscript. JW, XJ, FL, ChuW, QZ, and MM implemented the experiments. XM, YL, YHL, NL, and QW performed the data analysis. XW, YZ, YJ, WM, and JW reviewed and edited the manuscript. All authors contributed to the article and approved the submitted version.

## Supplementary material

Supplementary Table S1 The Firmicutes/Bacteroidetes ratio (F/B ratio) in different intestinal segments.

Supplementary Table S2 Changes in the abundance of the microbiota before and after CLP in the small intestine.

Supplementary Table S3 Changes in the abundance of the microbiota before and after CLP in the colon.

Supplementary Table S4 Changes in the abundance of the microbiota in different intestinal segments in the sham group.

Supplementary Table S5 Changes in the abundance of the microbiota in different intestinal segments in the CLP6h group.
