# Supplementary material for: Changes in the microbiota in different intestinal segments of mice with sepsis
Source: Front Cell Infect Microbiol. 2023 Jan 10;12:954347. doi: 10.3389/fcimb.2022.954347 (PMC9871835; doi:10.3389/fcimb.2022.954347)
Supplement: Supplementary Table S1 — The Firmicutes/Bacteroidetes ratio (F/B ratio) in different intestinal segments. [file Table_1.docx]

Table S1: The Firmicutes/Bacteroidetes ratio (F/B ratio) in different intestinal segments.

| Phylum | S.sham | C.sham | S.CLP6 | C.CLP6 |
| --- | --- | --- | --- | --- |
| Firmicutes | 95375.8 | 58967.9 | 75107.9 | 39867.9 |
| Bacteroidetes | 5695.7 | 34879.2 | 16020.8 | 44713.5 |
| Actinobacteria | 2808.9 | 1458 | 1549.4 | 172.7 |
| Verrucomicrobia | 338.3 | 1940.9 | 696.3 | 1241.3 |
| Proteobacteria | 171.7 | 379.1 | 1721.5 | 1590.9 |
| Epsilonbacteraeota | 3.7 | 185.6 | 5.3 | 1549.2 |
| Tenericutes | 18 | 359.1 | 852.9 | 342.8 |
| Deferribacteres | 2.8 | 32 | 2.9 | 1394.2 |
| Patescibacteria | 87.8 | 131.2 | 31.7 | 68.9 |
| (Unassigned) | 14.2 | 44.1 | 34.4 | 38.6 |
| Cyanobacteria | 19.7 | 13.5 | 0.9 | 18.9 |
| Firmicutes / Bacteroidetes ratio | 16.74522886 | 1.690632239 | 4.688149156 | 0.891630045 |
