# Supplementary material for: Changes in the microbiota in different intestinal segments of mice with sepsis
Source: Front Cell Infect Microbiol. 2023 Jan 10;12:954347. doi: 10.3389/fcimb.2022.954347 (PMC9871835; doi:10.3389/fcimb.2022.954347)
Supplement: Supplementary Table S2 — Changes in the abundance of the microbiota before and after CLP in the small intestine. [file Table_2.docx]

Table S2: Changes in the abundance of the microbiota before and after CLP in the small intestine.

| Taxon | S.CLP6_average | S.sham_average | S.CLP6_S.sham_diff | P value |
| --- | --- | --- | --- | --- |
| OTU_1:k:Bacteria,p:Firmicutes,c:Bacilli,o:Lactobacillales,f:Lactobacillaceae,g:Lactobacillus | 0.286808797 | 0.366698534 | 0.079889737 | 0.315789108 |
| OTU_2:k:Bacteria,p:Firmicutes,c:Bacilli,o:Lactobacillales,f:Lactobacillaceae,g:Lactobacillus | 0.283125867 | 0.099312993 | -0.183812873 | 0.999756436 |
| OTU_4:k:Bacteria,p:Firmicutes,c:Bacilli,o:Lactobacillales,f:Lactobacillaceae,g:Lactobacillus | 0.096635388 | 0.189727813 | 0.093092424 | 0.09678042 |
| OTU_5:k:Bacteria,p:Bacteroidetes,c:Bacteroidia,o:Bacteroidales,f:Muribaculaceae | 0.014307858 | 0.022117327 | 0.007809469 | 0.557381258 |
| OTU_12:k:Bacteria,p:Bacteroidetes,c:Bacteroidia,o:Bacteroidales,f:Muribaculaceae | 0.007319432 | 0.000473184 | -0.006846248 | 0.768477699 |
| OTU_15:k:Bacteria,p:Bacteroidetes,c:Bacteroidia,o:Bacteroidales,f:Muribaculaceae | 0.030973209 | 0.000269603 | -0.030703606 | 0.999756436 |
| OTU_10:k:Bacteria,p:Firmicutes,c:Erysipelotrichia,o:Erysipelotrichales,f:Erysipelotrichaceae,g:Dubosiella,s:Firmicutes_bacterium_M10-2 | 0.009336102 | 0.029895276 | 0.020559174 | 0.227851623 |
| OTU_19:k:Bacteria,p:Firmicutes,c:Erysipelotrichia,o:Erysipelotrichales,f:Erysipelotrichaceae,g:Allobaculum,s:uncultured_bacterium | 0.032998072 | 0.025920004 | -0.007078068 | 0.557381258 |
| OTU_17:k:Bacteria,p:Firmicutes,c:Erysipelotrichia,o:Erysipelotrichales,f:Erysipelotrichaceae,g:Allobaculum,s:uncultured_bacterium | 0.0001032 | 0.038447412 | 0.038344213 | 0.199826037 |
| OTU_27:k:Bacteria,p:Firmicutes,c:Erysipelotrichia,o:Erysipelotrichales,f:Erysipelotrichaceae,g:Faecalibaculum,s:uncultured_bacterium | 0.000179528 | 0.027732042 | 0.027552513 | 0.123801695 |
| OTU_9:k:Bacteria,p:Firmicutes,c:Bacilli,o:Lactobacillales,f:Lactobacillaceae,g:Lactobacillus | 0.012689294 | 0.027345642 | 0.014656348 | 0.154704515 |
| OTU_6:k:Bacteria,p:Firmicutes,c:Clostridia,o:Clostridiales,f:Clostridiaceae_1,g:Candidatus_Arthromitus | 0.025749184 | 0.018313501 | -0.007435683 | 0.905916555 |
| OTU_8:k:Bacteria,p:Bacteroidetes,c:Bacteroidia,o:Bacteroidales,f:Prevotellaceae,g:Alloprevotella,s:uncultured_Bacteroidales_bacterium | 0.001103594 | 6.82E-05 | -0.001035361 | 0.474826665 |
| OTU_11:k:Bacteria,p:Bacteroidetes,c:Bacteroidia,o:Bacteroidales,f:Muribaculaceae | 0.017452554 | 0.008129804 | -0.00932275 | 0.944932967 |
| OTU_7:k:Bacteria,p:Verrucomicrobia,c:Verrucomicrobiae,o:Verrucomicrobiales,f:Akkermansiaceae,g:Akkermansia | 0.007564165 | 0.003448611 | -0.004115554 | 0.852949834 |
| OTU_14:k:Bacteria,p:Bacteroidetes,c:Bacteroidia,o:Bacteroidales,f:Muribaculaceae | 0.022578647 | 5.37E-05 | -0.022524899 | 0.999756436 |
| OTU_37:k:Bacteria,p:Firmicutes,c:Clostridia,o:Clostridiales,f:Lachnospiraceae,g:Lachnospiraceae_NK4A136_group | 0.000571185 | 0.005315053 | 0.004743867 | 0.041554914 |
| OTU_13:k:Bacteria,p:Bacteroidetes,c:Bacteroidia,o:Bacteroidales,f:Muribaculaceae | 0.00848572 | 0.010900973 | 0.002415254 | 0.852332605 |
| OTU_29:k:Bacteria,p:Bacteroidetes,c:Bacteroidia,o:Bacteroidales,f:Muribaculaceae | 0.00564827 | 0.000232287 | -0.005415983 | 0.837133488 |
| OTU_25:k:Bacteria,p:Bacteroidetes,c:Bacteroidia,o:Bacteroidales,f:Muribaculaceae | 0.001207378 | 0.000130517 | -0.00107686 | 0.837133488 |
