# Supplementary material for: Changes in the microbiota in different intestinal segments of mice with sepsis
Source: Front Cell Infect Microbiol. 2023 Jan 10;12:954347. doi: 10.3389/fcimb.2022.954347 (PMC9871835; doi:10.3389/fcimb.2022.954347)
Supplement: Supplementary Table S3 — Changes in the abundance of the microbiota before and after CLP in the colon. [file Table_3.docx]

Table S3: Changes in the abundance of the microbiota before and after CLP in the colon.

| Taxon | C.CLP6_average | C.sham_average | C.CLP6_C.sham_diff | P value |
| --- | --- | --- | --- | --- |
| OTU_1:k:Bacteria,p:Firmicutes,c:Bacilli,o:Lactobacillales,f:Lactobacillaceae,g:Lactobacillus | 0.024428451 | 0.137925468 | 0.113497017 | 0.060312834 |
| OTU_2:k:Bacteria,p:Firmicutes,c:Bacilli,o:Lactobacillales,f:Lactobacillaceae,g:Lactobacillus | 0.007760323 | 0.072720743 | 0.06496042 | 0.000906601 |
| OTU_4:k:Bacteria,p:Firmicutes,c:Bacilli,o:Lactobacillales,f:Lactobacillaceae,g:Lactobacillus | 0.006047409 | 0.053240764 | 0.047193355 | 0.005439607 |
| OTU_5:k:Bacteria,p:Bacteroidetes,c:Bacteroidia,o:Bacteroidales,f:Muribaculaceae | 0.018829203 | 0.07568007 | 0.056850867 | 0.021800046 |
| OTU_12:k:Bacteria,p:Bacteroidetes,c:Bacteroidia,o:Bacteroidales,f:Muribaculaceae | 0.067764324 | 0.042033581 | -0.025730743 | 1 |
| OTU_15:k:Bacteria,p:Bacteroidetes,c:Bacteroidia,o:Bacteroidales,f:Muribaculaceae | 0.044288124 | 0.016653837 | -0.027634286 | 1 |
| OTU_10:k:Bacteria,p:Firmicutes,c:Erysipelotrichia,o:Erysipelotrichales,f:Erysipelotrichaceae,g:Dubosiella,s:Firmicutes_bacterium_M10-2 | 0.00022474 | 0.046196927 | 0.045972187 | 0.074803403 |
| OTU_19:k:Bacteria,p:Firmicutes,c:Erysipelotrichia,o:Erysipelotrichales,f:Erysipelotrichaceae,g:Allobaculum,s:uncultured_bacterium | 0.000127064 | 0.013208798 | 0.013081734 | 0.26261213 |
| OTU_17:k:Bacteria,p:Firmicutes,c:Erysipelotrichia,o:Erysipelotrichales,f:Erysipelotrichaceae,g:Allobaculum,s:uncultured_bacterium | 5.17E-05 | 0.022538169 | 0.02248644 | 0.176720885 |
| OTU_27:k:Bacteria,p:Firmicutes,c:Erysipelotrichia,o:Erysipelotrichales,f:Erysipelotrichaceae,g:Faecalibaculum,s:uncultured_bacterium | 8.56E-05 | 0.02303869 | 0.022953087 | 0.074803403 |
| OTU_9:k:Bacteria,p:Firmicutes,c:Bacilli,o:Lactobacillales,f:Lactobacillaceae,g:Lactobacillus | 0.000864068 | 0.007627261 | 0.006763192 | 0.010105481 |
| OTU_6:k:Bacteria,p:Firmicutes,c:Clostridia,o:Clostridiales,f:Clostridiaceae_1,g:Candidatus_Arthromitus | 0.000643768 | 0.003110783 | 0.002467015 | 0.010105481 |
| OTU_8:k:Bacteria,p:Bacteroidetes,c:Bacteroidia,o:Bacteroidales,f:Prevotellaceae,g:Alloprevotella,s:uncultured_Bacteroidales_bacterium | 0.041969902 | 0.007325104 | -0.034644798 | 1 |
| OTU_11:k:Bacteria,p:Bacteroidetes,c:Bacteroidia,o:Bacteroidales,f:Muribaculaceae | 0.019169349 | 0.00363706 | -0.015532289 | 1 |
| OTU_7:k:Bacteria,p:Verrucomicrobia,c:Verrucomicrobiae,o:Verrucomicrobiales,f:Akkermansiaceae,g:Akkermansia | 0.014872804 | 0.019268624 | 0.00439582 | 0.979920669 |
| OTU_14:k:Bacteria,p:Bacteroidetes,c:Bacteroidia,o:Bacteroidales,f:Muribaculaceae | 0.022190503 | 0.000264623 | -0.02192588 | 1 |
| OTU_37:k:Bacteria,p:Firmicutes,c:Clostridia,o:Clostridiales,f:Lachnospiraceae,g:Lachnospiraceae_NK4A136_group | 0.017512453 | 0.019043281 | 0.001530828 | 1 |
| OTU_13:k:Bacteria,p:Bacteroidetes,c:Bacteroidia,o:Bacteroidales,f:Muribaculaceae | 0.015702246 | 0.006632125 | -0.009070121 | 1 |
| OTU_29:k:Bacteria,p:Bacteroidetes,c:Bacteroidia,o:Bacteroidales,f:Muribaculaceae | 0.016217302 | 0.012814818 | -0.003402484 | 1 |
| OTU_25:k:Bacteria,p:Bacteroidetes,c:Bacteroidia,o:Bacteroidales,f:Muribaculaceae | 0.019321294 | 0.010575639 | -0.008745655 | 1 |
