# Supplementary material for: Changes in the microbiota in different intestinal segments of mice with sepsis
Source: Front Cell Infect Microbiol. 2023 Jan 10;12:954347. doi: 10.3389/fcimb.2022.954347 (PMC9871835; doi:10.3389/fcimb.2022.954347)
Supplement: Supplementary Table S4 — Changes in the abundance of the microbiota in different intestinal segments in the sham group. [file Table_4.docx]

Table S4: Changes in the abundance of the microbiota in different intestinal segments in the sham group.

| Taxon | C.sham_average | S.sham_average | C.sham_S.sham_diff | p value |
| --- | --- | --- | --- | --- |
| OTU_1:k:Bacteria,p:Firmicutes,c:Bacilli,o:Lactobacillales,f:Lactobacillaceae,g:Lactobacillus | 0.137925468 | 0.366698534 | 0.228773066 | 0.160770602 |
| OTU_2:k:Bacteria,p:Firmicutes,c:Bacilli,o:Lactobacillales,f:Lactobacillaceae,g:Lactobacillus | 0.072720743 | 0.099312993 | 0.02659225 | 1 |
| OTU_4:k:Bacteria,p:Firmicutes,c:Bacilli,o:Lactobacillales,f:Lactobacillaceae,g:Lactobacillus | 0.053240764 | 0.189727813 | 0.136487049 | 0.160770602 |
| OTU_5:k:Bacteria,p:Bacteroidetes,c:Bacteroidia,o:Bacteroidales,f:Muribaculaceae | 0.07568007 | 0.022117327 | -0.053562744 | 1 |
| OTU_12:k:Bacteria,p:Bacteroidetes,c:Bacteroidia,o:Bacteroidales,f:Muribaculaceae | 0.042033581 | 0.000473184 | -0.041560397 | 1 |
| OTU_15:k:Bacteria,p:Bacteroidetes,c:Bacteroidia,o:Bacteroidales,f:Muribaculaceae | 0.016653837 | 0.000269603 | -0.016384234 | 1 |
| OTU_10:k:Bacteria,p:Firmicutes,c:Erysipelotrichia,o:Erysipelotrichales,f:Erysipelotrichaceae,g:Dubosiella,s:Firmicutes_bacterium_M10-2 | 0.046196927 | 0.029895276 | -0.016301651 | 1 |
| OTU_19:k:Bacteria,p:Firmicutes,c:Erysipelotrichia,o:Erysipelotrichales,f:Erysipelotrichaceae,g:Allobaculum,s:uncultured_bacterium | 0.013208798 | 0.025920004 | 0.012711206 | 1 |
| OTU_17:k:Bacteria,p:Firmicutes,c:Erysipelotrichia,o:Erysipelotrichales,f:Erysipelotrichaceae,g:Allobaculum,s:uncultured_bacterium | 0.022538169 | 0.038447412 | 0.015909244 | 1 |
| OTU_27:k:Bacteria,p:Firmicutes,c:Erysipelotrichia,o:Erysipelotrichales,f:Erysipelotrichaceae,g:Faecalibaculum,s:uncultured_bacterium | 0.02303869 | 0.027732042 | 0.004693352 | 1 |
| OTU_9:k:Bacteria,p:Firmicutes,c:Bacilli,o:Lactobacillales,f:Lactobacillaceae,g:Lactobacillus | 0.007627261 | 0.027345642 | 0.019718381 | 0.320936803 |
| OTU_6:k:Bacteria,p:Firmicutes,c:Clostridia,o:Clostridiales,f:Clostridiaceae_1,g:Candidatus_Arthromitus | 0.003110783 | 0.018313501 | 0.015202719 | 0.603116408 |
| OTU_8:k:Bacteria,p:Bacteroidetes,c:Bacteroidia,o:Bacteroidales,f:Prevotellaceae,g:Alloprevotella,s:uncultured_Bacteroidales_bacterium | 0.007325104 | 6.82E-05 | -0.00725687 | 1 |
| OTU_11:k:Bacteria,p:Bacteroidetes,c:Bacteroidia,o:Bacteroidales,f:Muribaculaceae | 0.00363706 | 0.008129804 | 0.004492744 | 1 |
| OTU_7:k:Bacteria,p:Verrucomicrobia,c:Verrucomicrobiae,o:Verrucomicrobiales,f:Akkermansiaceae,g:Akkermansia | 0.019268624 | 0.003448611 | -0.015820012 | 1 |
| OTU_14:k:Bacteria,p:Bacteroidetes,c:Bacteroidia,o:Bacteroidales,f:Muribaculaceae | 0.000264623 | 5.37E-05 | -0.000210875 | 1 |
| OTU_37:k:Bacteria,p:Firmicutes,c:Clostridia,o:Clostridiales,f:Lachnospiraceae,g:Lachnospiraceae_NK4A136_group | 0.019043281 | 0.005315053 | -0.013728229 | 1 |
| OTU_13:k:Bacteria,p:Bacteroidetes,c:Bacteroidia,o:Bacteroidales,f:Muribaculaceae | 0.006632125 | 0.010900973 | 0.004268849 | 1 |
| OTU_29:k:Bacteria,p:Bacteroidetes,c:Bacteroidia,o:Bacteroidales,f:Muribaculaceae | 0.012814818 | 0.000232287 | -0.01258253 | 1 |
| OTU_25:k:Bacteria,p:Bacteroidetes,c:Bacteroidia,o:Bacteroidales,f:Muribaculaceae | 0.010575639 | 0.000130517 | -0.010445122 | 1 |
