# Supplementary material for: Changes in the microbiota in different intestinal segments of mice with sepsis
Source: Front Cell Infect Microbiol. 2023 Jan 10;12:954347. doi: 10.3389/fcimb.2022.954347 (PMC9871835; doi:10.3389/fcimb.2022.954347)
Supplement: Supplementary Table S5 — Changes in the abundance of the microbiota in different intestinal segments in the CLP6h group. [file Table_5.docx]

Table S5: Changes in the abundance of the microbiota in different intestinal segments in the CLP6h group.

| Taxon | C.CLP6_average | S.CLP6_average | C.CLP6_S.CLP6_diff | p value |
| --- | --- | --- | --- | --- |
| OTU_1:k:Bacteria,p:Firmicutes,c:Bacilli,o:Lactobacillales,f:Lactobacillaceae,g:Lactobacillus | 0.024428451 | 0.286808797 | 0.262380346 | 0.000903895 |
| OTU_2:k:Bacteria,p:Firmicutes,c:Bacilli,o:Lactobacillales,f:Lactobacillaceae,g:Lactobacillus | 0.007760323 | 0.283125867 | 0.275365544 | 0.000903895 |
| OTU_4:k:Bacteria,p:Firmicutes,c:Bacilli,o:Lactobacillales,f:Lactobacillaceae,g:Lactobacillus | 0.006047409 | 0.096635388 | 0.090587979 | 0.013558423 |
| OTU_5:k:Bacteria,p:Bacteroidetes,c:Bacteroidia,o:Bacteroidales,f:Muribaculaceae | 0.018829203 | 0.014307858 | -0.004521346 | 1 |
| OTU_12:k:Bacteria,p:Bacteroidetes,c:Bacteroidia,o:Bacteroidales,f:Muribaculaceae | 0.067764324 | 0.007319432 | -0.060444892 | 1 |
| OTU_15:k:Bacteria,p:Bacteroidetes,c:Bacteroidia,o:Bacteroidales,f:Muribaculaceae | 0.044288124 | 0.030973209 | -0.013314915 | 1 |
| OTU_10:k:Bacteria,p:Firmicutes,c:Erysipelotrichia,o:Erysipelotrichales,f:Erysipelotrichaceae,g:Dubosiella,s:Firmicutes_bacterium_M10-2 | 0.00022474 | 0.009336102 | 0.009111362 | 0.040072672 |
| OTU_19:k:Bacteria,p:Firmicutes,c:Erysipelotrichia,o:Erysipelotrichales,f:Erysipelotrichaceae,g:Allobaculum,s:uncultured_bacterium | 0.000127064 | 0.032998072 | 0.032871008 | 0.437756284 |
| OTU_17:k:Bacteria,p:Firmicutes,c:Erysipelotrichia,o:Erysipelotrichales,f:Erysipelotrichaceae,g:Allobaculum,s:uncultured_bacterium | 5.17E-05 | 0.0001032 | 5.15E-05 | 1 |
| OTU_27:k:Bacteria,p:Firmicutes,c:Erysipelotrichia,o:Erysipelotrichales,f:Erysipelotrichaceae,g:Faecalibaculum,s:uncultured_bacterium | 8.56E-05 | 0.000179528 | 9.39E-05 | 0.087886393 |
| OTU_9:k:Bacteria,p:Firmicutes,c:Bacilli,o:Lactobacillales,f:Lactobacillaceae,g:Lactobacillus | 0.000864068 | 0.012689294 | 0.011825226 | 0.034890342 |
| OTU_6:k:Bacteria,p:Firmicutes,c:Clostridia,o:Clostridiales,f:Clostridiaceae_1,g:Candidatus_Arthromitus | 0.000643768 | 0.025749184 | 0.025105416 | 0.040072672 |
| OTU_8:k:Bacteria,p:Bacteroidetes,c:Bacteroidia,o:Bacteroidales,f:Prevotellaceae,g:Alloprevotella,s:uncultured_Bacteroidales_bacterium | 0.041969902 | 0.001103594 | -0.040866308 | 1 |
| OTU_11:k:Bacteria,p:Bacteroidetes,c:Bacteroidia,o:Bacteroidales,f:Muribaculaceae | 0.019169349 | 0.017452554 | -0.001716795 | 1 |
| OTU_7:k:Bacteria,p:Verrucomicrobia,c:Verrucomicrobiae,o:Verrucomicrobiales,f:Akkermansiaceae,g:Akkermansia | 0.014872804 | 0.007564165 | -0.007308638 | 1 |
| OTU_14:k:Bacteria,p:Bacteroidetes,c:Bacteroidia,o:Bacteroidales,f:Muribaculaceae | 0.022190503 | 0.022578647 | 0.000388144 | 1 |
| OTU_37:k:Bacteria,p:Firmicutes,c:Clostridia,o:Clostridiales,f:Lachnospiraceae,g:Lachnospiraceae_NK4A136_group | 0.017512453 | 0.000571185 | -0.016941268 | 1 |
| OTU_13:k:Bacteria,p:Bacteroidetes,c:Bacteroidia,o:Bacteroidales,f:Muribaculaceae | 0.015702246 | 0.00848572 | -0.007216526 | 1 |
| OTU_29:k:Bacteria,p:Bacteroidetes,c:Bacteroidia,o:Bacteroidales,f:Muribaculaceae | 0.016217302 | 0.00564827 | -0.010569032 | 1 |
| OTU_25:k:Bacteria,p:Bacteroidetes,c:Bacteroidia,o:Bacteroidales,f:Muribaculaceae | 0.019321294 | 0.001207378 | -0.018113917 | 1 |
